# Robust Transmit Antenna Design for Performance Improvement of Cell-Edge Users: Approach of NOMA and Outage/Ergodic Capacity Analysis

**DOI:** 10.3390/s19224907

**Published:** 2019-11-10

**Authors:** Dinh-Thuan Do, Chi-Bao Le, Byung Moo Lee

**Affiliations:** 1 Wireless Communications Research Group, Faculty of Electrical and Electronics Engineering, Ton Duc Thang University, Ho Chi Minh City 700000, Vietnam; 2 Faculty of Electronics Technology, Industrial University of Ho Chi Minh City (IUH), Ho Chi Minh City 700000, Vietnam; lechibao@iuh.edu.vn; 3 School of Intelligent Mechatronics Engineering, Sejong University, Seoul 05006, Korea

**Keywords:** non-orthogonal multiple access, outage probability, wireless sensor network, transmit antenna selection, ergodic capacity

## Abstract

In this investigation, a wireless sensor network using a non-orthogonal multiple access (NOMA) system is considered in two scenarios related to the number of serving access point/base stations, where two policies provide system performance improvement in two sensors (the near user and the far user). To improve performance efficiency, two robust transmit antenna strategies are designed related to the access point/base station (BS), namely (i) Transmit Antenna Selection (TAS) mode and (ii) two base station (TBS) approach to simultaneously serve NOMA users. First, the TAS scheme is implemented to provide suboptimal outage performance for such NOMA, in which BS equipped at least two antennas while NOMA users are equippeda single antenna. Secondly, the TBS scheme is conducted to enhance the outage performance, especially considering priority evaluation for the far user in user pairs. As an important result, such far users in two proposed schemes are studied by introducing the exact closed-form expression to examine outage behavior. Accordingly, the closed-form expressions regarding ergodic capacity can be further obtained. To corroborate the exactness of these metrics, Monte Carlo simulation is performed. In addition, the proposed schemes exhibit various performance evaluations accompanied by different related parameters such as power allocation factors, the number of transmit antenna, and transmit signal-to-noise ratio (SNR).

## 1. Introduction

Towards fifth-generation (5G) wireless communication, mobile networks have attracted a huge amount of research with a major increase in recent years. First, spectral and energy efficiency are two folds of the enhancing system which aims to introduce strategy to fulfill 5G requirements and adapt fast speed in data transmission with thousand-fold higher rates [1,2]. Secondly, massive connections are acquired in wireless sensor networks. In particular, to satisfy high spectral efficiency, a potential band-efficient methodology, namely non-orthogonal multiple access (NOMA), is established for the future mobile networks [3,4,5,6,7]. To serve multiple users, superposition coding designed in NOMA systems is deployed at the transmitter side, while detecting signal operation is performed by successive interference cancellation (SIC) technology implemented at the receiver side. The distinguished transmission power of the signals is allocated for a group of NOMA users who occupy dissimilar channel gains. Such interesting characterization means that with the same time/frequency/code, multiple users can access to the BS simultaneously. Comparing the orthogonal multiple access (OMA), significantly superior performance of NOMA technology can be observed, and these related metrics can be evaluated such as the outage event and ergodic capacity as considering a downlink NOMA system [8]. In addition, a high fairness constraint together with suitable power allocation strategy is introduced in NOMA systems to guarantee the performance in the other model of NOMA system as in [9]. The authors in [10] showed stochastic geometry as a tool to derive the closed-form expressions of outage probability to perform the performance consideration on cognitive radio network which operates as an underlay NOMA. In the other system model, the exact expression and bound of outage probability and ergodic sum rate in closed form are calculated with respect to performance evaluation of cooperative NOMA system using an amplify and forward (AF) via Nakagami-m fading channels as investigation in [11]. Recently, relaying schemes [12] have been combined to implement cooperative NOMA networks [13,14] thanks to two main advantages of reliable and higher coverage area from relaying.

Regarding the wireless channel, the sequence of the instantaneous channel gain or exact channel state information (CSI) at the transmitter side have been supposed in many of the recent works related to NOMA [15]. However, underwater acoustics [16] and high-speed railway (HSR) [17] systems with a quickly varying channel and the large feedback delays lead to the assumption of perfect CSI becoming nearly impractical for many communication scenarios. Recently, NOMA systems with statistical CSI have been considered in recent works [16,18]. In [19], they considered multicarrier NOMA systems with a power-efficient resource distribution structure in terms of statistical CSI. Meanwhile, orthogonal frequency-division multiplexing (OFDM) systems have been investigated in the downlink of underwater acoustic networks for NOMA [16]. By deploying statistical CSI and changing power distribution for dissimilar users, the transmitter can modify the system’s sum throughput in NOMA schemes, but in this case perfect CSI is assumed at the transmitter side. Additionally, as reported in [20], more antenna assigned at the BS resulted in improvement at the cell-center user. Hence, it may harm the quality of service (QoS) of the cell-center users since a major part of the power budget is allocated to cell-edge users, otherwise, it may compromise the reception reliability of the cell-edge users [21].

Due to the flexibility and energy efficiency, wireless-powered relaying communication [22,23,24,25,26] provides further advantages to the deployment of NOMA. This combination is deployed in many other wireless technologies to improve system performance, such as [27]. Recently, to further improve system performance, devices equipped with multiple antennae have been exploited in NOMA systems [28,29,30]. As an important advantage, multiple-input multiple-output (MIMO) NOMA systems provide improved performance in terms of the sum rate, and it is strictly larger compared with the MIMO OMA system in [29]. A multiple-antenna energy-harvesting relay is examined in terms of outage performance in the NOMA system as in [30]. In such MIMO NOMA, the number of antennae exhibit potential in performance improvement. However, such improvement requires expensive RF chains at the terminal. Fortunately, transmit antenna selection (TAS) technique has been proposed to avoid the high hardware costs while preserving benefits from multiple antennae design with respect to the enhanced diversity and throughput [31]. Furthermore, other novel applications of NOMA can be seen in emerging techniques such as millimeter wave [32] and visible light communication [33], etc.

In this investigation, motivated by novel results from [20,21,28] we propose two policies for wireless sensor networks related to different transmission modes. In fact, due to higher cost of deployment of system reported in [28], the proposed model in this paper is simpler than that in [28]. In particular, this paper considers one policy following TAS scheme while another requires two BSs to simultaneous serve users. Two such BSs need synchronous procedures from the base station controller to transmit a superimposed signal. Such modes can be applied in the relaying communications to show advantages of both enhanced reliability in relaying and massive connection in NOMA strategy. To the best of our knowledge, there are very few studies that compare between the TAS and two simultaneous transmissions of link BS-user with respect to outage and ergodic capacity performance in the existing literature. To provide a more specific system, we study the downlink NOMA which comprises of one/two BS serving multiple users. For efficient and tractable computation, we consider a two-antenna model in such a NOMA network. Under these concerns, we achieve the following main results in our paper:

- At a glance, the first main contribution shows the advanced deployment of one/two base stations (access points) to serve destination users on the downlink in NOMA. This study proposes to jointly obtain the advantage of the superimposed signal and multiple access scheme to satisfy fairness data rate of the far user together with its improved outage performance.
- We show that the considered approaches effectively improve the far user’s outage behavior, which results from both transmit antenna selection decision regime and the cooperative NOMA scheme. However, the design of TAS (Scheme I) is a difficult task. To address such a disadvantage, Scheme II provides a simpler process than Scheme I but at least two nearby base stations (access points) are strictly acquired in the synchronization process at the receiver in Scheme II due to implement two BSs serving the far NOMA user in such case. Such nearby BS architecture is motivated by works in [34], or beamforming antenna design in [35]. It can be confirmed that the higher diversity can be achieved with a lower cost in design.
- As a third contribution, simulation and analytical results corroborate the exactness of our derived expressions and the advantage of NOMA. Furthermore, the outage performance in two schemes can be seen in opposite trends of the ergodic performance. In particular, Scheme I has better outage performance, while worse ergodic capacity can be achieved in Scheme I as compared with those in Scheme II.
- To provide a valuable benchmark, imperfect CSI is further studied, and degradation performance can worsen. The amount of channel error is controlled to maintain an acceptable outage performance. In most of the papers related to NOMA, two users are studied, but this paper further examines how system performance changes in scenarios where multiple users are served. The outage performance is further evaluated in Scheme III.

The rest of this study is presented as follows. Section 2 presents the proposed schemes with the corresponding system model and designates aspects related to wireless signal processing. Section 3 shows the performance examinations in terms of the outage and ergodic capacity for the considered scenarios in Scheme I, and then it is compared with the remaining architecture of MISO NOMA presented in Section 4. The multiple NOMA users can be evaluated in Scheme III at Section 5. The insightful discussions are provided in Section 6, which further presents some illustrative numerical results. To corroborate the concerned analysis, Monte Carlo simulations are performed. Finally, several remarks and a conclusion are presented in Section 7 to show the important points of the paper.

**Notations**: To simplify illustrations of expressions, $\mathrm{Pr}\left[.\right]$ symbolizes probability, the probability density function (PDF) and the cumulative distribution function (CDF) of a random variable *X* are represented as $F_{X}\left(.\right)$ and $f_{x}\left(.\right)$, respectively, $Ei\left(.\right)$ is incomplete gamma function and $E\left[.\right]$ stands for the expectation operator.

## 2. System Model

**Figure 1:**
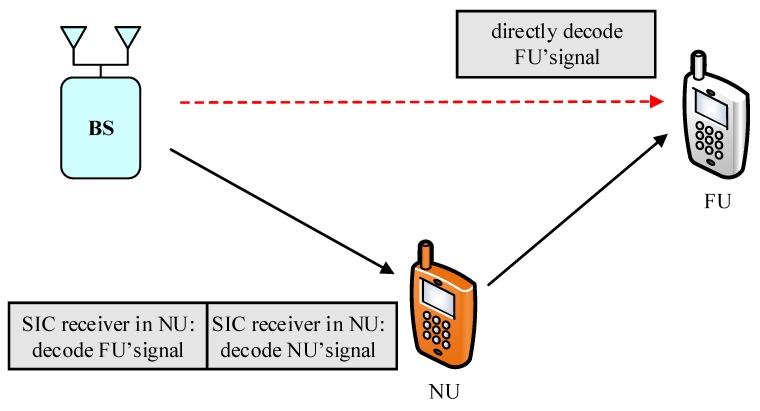
System model of MISO NOMA with antenna selection at the BS.

We consider cooperative Decode and Forward (DF) relaying network, where the base station (BS) communicates with two NOMA devices consisting of the near user and the far user by requiring the help of an intermediate relay $\left(R\right)$. These approaches are presented in two schemes. The link between the source and the destination is unreliable or unavailable, so the transmission can only happen successfully with the aid of a relay. The interesting thing here is that the relay is the fixed power-based equipment. As a result, this paper replaces the system model presented in [20,21] due to small amounts of harvested power to the feed operation of the relay and complex computation. In particular, the relay node deployed in this paper is characterized as an individual power to guarantee its own signal processing. Furthermore, each node is a furnished single antenna, and half-duplex mode using the DF strategy, which is conventionally deployed in the relay. The relay in Figure 1 for Scheme I acquires two independent data symbols during two time epochs, $x_{FU}$ and $x_{NU}$ transmitted from the BS directly, and such signal processing needs the assistance of the relay, whereas the NOMA relaying scheme delivers data symbols with more chance of antenna selection.

**Figure 2:**
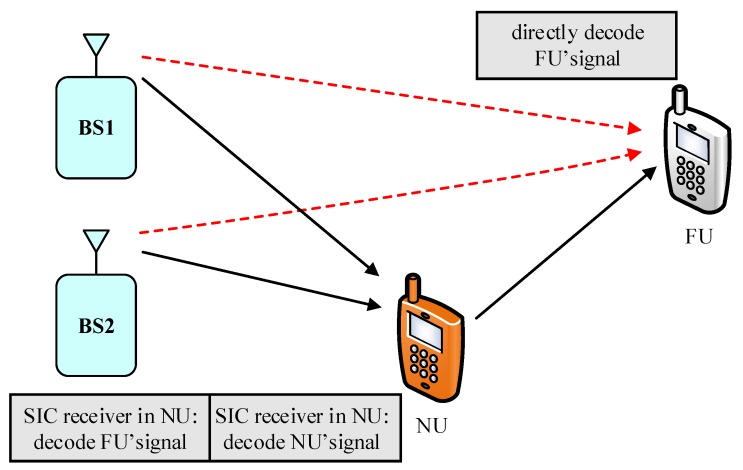
System model for D2D NOMA network.

As illustrated in Figure 1, we examine a MISO-NOMA downlink transmission where a BS is required to serve a two-user NOMA. The BS is assigned antenna architecture to simultaneously communicate with a near user, named User NU, and a far user, known as User FU. In the design of TAS scheme, the BS is equipped with only two antennae, while each user is equipped with single antenna. According to the NOMA principle, the BS simultaneously transmits signal $\sqrt{\phi_{2}P_{S}}x_{NU}+\sqrt{\phi_{1}P_{S}}x_{FU}$ to all the users. While two single antenna BSs are required to simultaneous serve FU and NU user as in Figure 2, each BS in Scheme II transmits $\sqrt{\frac{\phi_{2}P_{S}}{2}}x_{NU}+\sqrt{\frac{\phi_{1}P_{S}}{2}}x_{FU}$. In these cases, $x_{NU},x_{FU}$ are assigned for User NU and FU, respectively. $P_{S}$ stands for transmit power at the base station. It is further assumed that power coefficients $\phi_{1},\phi_{2}$ are allocated for User NU and FU, respectively. It is assumed that $\phi_{2}<\phi_{1}$ and it satisfies $\phi_{2}+\phi_{1}=1$. The wireless channels proceed signal on each link following Rayleigh fading and are affected by additive white Gaussian noise (AWGN) at receivers. In addition, the links BS-NU, NU-FU, BS-FU are represented by channel coefficients with denotations of $h_{iN},h_{NF},h_{iF}\left(i=1,2\right)$. These channel coefficients corresponding to such links are the independent Rayleigh random variables with zero mean together with variances of $\lambda_{iN},\Omega_{NF},\Omega_{iF}$, respectively. Hence, the channel gains ${\left|h_{iN}\right|}^{2},{\left|h_{NF}\right|}^{2},{\left|h_{iF}\right|}^{2}$ are exponentially distributed random variables with mean values as concerns $\Omega_{iN},\Omega_{NF},\Omega_{iF}$. It is denoted that $\Omega_{n}=d_{n}^{-\mu }$ to display the preferred effect of the distance between these nodes, where μ is the path loss exponent and $d_{n}$ is distance of related link. The concerned distance values are normalized to the unit for ease of computation.

As a main contribution, we determine the outage performance and ergodic capacity of NOMA relaying networks in the presence of one BS/two BSs approaches. Specifically, TAS and TBS techniques are exploited at the multi-antenna base station or two simultaneous serving BSs to enhance the quality of transmission employing NOMA. It is worth noting that the transmit antenna scheme requires selection combining (SC) for the receiver. In the proposed TBS, two BSs send superimposed signals to the User NU and the User FU at the same time and hence both NU and FU user must be able to combine signal from two links. To study system performance, we first derive several expressions of signal-to-noise ratio (SNR) and further investigate the outage behavior and ergodic capacity. More specifically, TAS scheme supposes that antenna *i* is selected on the BS for effective information transmission to User FU and User NU who follow the principle of NOMA transmission, but the FU needs higher priority in performance evaluation due to far distance. In particular, users with better channel conditions in NOMA obtain less transmit power while more transmit power goes to users with worse channel conditions to balance advantages of the system throughput and user fairness. Thus, users with better channel conditions required to decode the signals for the others before decoding their own and hence the optimal order for SIC is in the order of the increasing channel gain.

## 3. Scheme I: Transmit Antenna Selection

Under NOMA scheme, the received signal based on observation of the selected antenna at User NU can be written as

(1) $$y_{iN}=\left(\sqrt{\phi_{2}P_{S}}x_{NU}+\sqrt{\phi_{1}P_{S}}x_{FU}\right)h_{iN}+n_{N},$$

where $n_{N}$ is complex additive white Gaussian noise (AWGN) with zero mean and variance $\sigma^{2}$. For ease of computation, AWGN noise terms at NU and FU are assumed equal, i.e., $\sigma^{2}$. It is noted that the SIC receiver at User NU first decodes $x_{FU}$ and then its own signal $x_{NU}$. To isolate superimposed symbols, SIC will be executed at each user as decoding processing at each user. The main advantage in NOMA is that it achieves detached signal by implementation of SIC, in which NOMA allocates less transmit power to users with better channel conditions and more transmit power to users with worse channel conditions in order. In particular, the received signal-to-interference-plus-noise ratio (SINR) at User NU to decode $x_{FU}$ can be first determined as

(2) $$\gamma_{iN,x_{FU}}^{}=\frac{\phi_{1}\rho {\left|h_{iN}\right|}^{2}}{\phi_{2}\rho {\left|h_{iN}\right|}^{2}+1},$$

where $\rho =\frac{P_{S}}{\sigma^{2}}$ is the transmit SNR. According to the principle of NOMA, the received SNR at User NU after subtracting interference component from the received signal to detect its own message to decode $x_{NU}$ can be computed by

(3) $$\gamma_{iN,x_{NU}}^{}=\phi_{2}\rho {\left|h_{iN}\right|}^{2},$$

In contrast with User NU, it is assumed that User FU can straight decode its information signal. The main reason to perform such an operation is that main signal in User FU is assigned with higher transmit power while the information signal of User NU produces the interference measured as noise. As a result, the received observation at User FU can be formulated by

(4) $$y_{iF}=\left(\sqrt{\phi_{2}P_{S}}x_{NU}+\sqrt{\phi_{1}P_{S}}x_{FU}\right)h_{iF}+n_{F},$$

Then, the received SNR at User FU is given by

(5) $$\gamma_{iF}=\frac{\phi_{1}\rho {\left|h_{iF}\right|}^{2}}{\phi_{2}\rho {\left|h_{iF}\right|}^{2}+1}.$$

By employing DF scheme at NU, ${\overset{⌢}{\;x}}_{FU}$ is the signal after the decoding procedure and then is forwarded to the User FU. We further obtain the received signal at FU as below

(6) $$y_{NF}=\sqrt{P_{N}}h_{NF}{\overset{⌢}{\;x}}_{FU}+n_{F}$$

where $n_{F}$ is complex additive white Gaussian noise (AWGN) with zero mean and variance $\sigma_{F}^{2}$. Next, the received SNR at User FU to detect $x_{FU}$ transmitted by User NU can be expressed by

(7) $$\gamma_{NF}=\rho {\left|h_{NF}\right|}^{2}.$$

Lastly, User FU combines two signals using a selection combining (SC) technique from two links, i.e., the direct link in which signal is received from the BS and the relay link in which signal is received from User NU. Thus, the achievable SNR after combining signal in two links corresponding with two received signals at User FU can be formulated as

(8) $$\gamma_{F}^{SC}=\max\left\{\gamma_{iF},\gamma_{NF}\right\}.$$

The end-to-end SNR to evaluate the received signal at the User FU is given by

(9) $$\gamma_{F}^{e2e}=\min\left\{\gamma_{iN,x_{F}}^{},\gamma_{F}^{SC}\right\}.$$

As a result, such equation yields to simple expression as

(10) $$\gamma_{F}^{e2e}=\min\left\{\gamma_{iN,x_{F}}^{},\max\left\{\gamma_{iF},\gamma_{NF}\right\}\right\}.$$

It is worth noting that regarding the complex constraint on $x_{FU}$, the signal $x_{FU}$ of User FU requires detection not only at User FU but also performed at the receiver of User NU with SIC. Thus, associating with selected antenna *i* the instantaneous rate obtained at User FU as

(11) $$\begin{array}{c} R_{iF}^{\left(1\right)}=\min\left\{\frac{1}{2}\log_{2}\left(1+\gamma_{iN,x_{F}}^{}\right),\frac{1}{2}\log_{2}\left(1+\gamma_{iF}\right)\right\}\\ \;\;\;\;\;\;\;\;\;\;\;\;\;=\frac{1}{2}\log_{2}\left(1+\min\left\{\frac{\phi_{1}\rho {\left|h_{iN}\right|}^{2}}{\phi_{2}\rho {\left|h_{iN}\right|}^{2}+1},\frac{\phi_{1}\rho {\left|h_{iF}\right|}^{2}}{\phi_{2}\rho {\left|h_{iF}\right|}^{2}+1}\right\}\right). \end{array}$$

It is noted that User NU has a shorter distance with the BS compared with User FU and hence ${\left|h_{iN}\right|}^{2}>{\left|h_{iF}\right|}^{2}$, we have

(12) $$R_{iF}^{\left(1\right)}=\frac{1}{2}\log_{2}\left(1+\frac{\phi_{1}\rho {\left|h_{iF}\right|}^{2}}{\phi_{2}\rho {\left|h_{iF}\right|}^{2}+1}\right).$$

A specific look on antenna criteria, to select an antenna that maximizes $R_{iF}^{\left(1\right)}$, we should apply the new TAS criterion adopted in Scheme I, and then it is illustrated as below

(13) $$\begin{array}{c} i^{*}=\arg\max_{1\leq i\leq K}\frac{1}{2}\log_{2}\left(1+\frac{\phi_{1}\rho {\left|h_{iF}\right|}^{2}}{\phi_{2}\rho {\left|h_{iF}\right|}^{2}+1}\right)\\ \;\;\;\;\;\;=\arg\max_{i\leq i\leq K}\frac{\phi_{1}\rho {\left|h_{iF}\right|}^{2}}{\phi_{2}\rho {\left|h_{iF}\right|}^{2}+1}\\ \;\;\;\;\;\;=\arg\max_{i\leq i\leq K}{\left|h_{iF}\right|}^{2} \end{array}$$

where *K* is the number of antennae at the BS in general case.

### 3.1. Outage Probability Analysis

The following section introduces an exact expression for the outage probability considering on the whole range of SNR and arbitrary path loss factor for NOMA systems, with given power allocation coefficients in NOMA. Mathematically, the achievable end-to-end rate for User FU can be computed as

(14) $$R_{iF}^{e2e}=\left\{\begin{array}{c} \frac{1}{2}\log_{2}\left(1+\gamma_{iF}\right),\;\;\mathrm{if}\;\;\;R_{iN}^{x_{FU}}<R_{2},\\ \frac{1}{2}\log_{2}\left(1+\gamma_{F}^{SC}\right),\;\;\mathrm{if}\;\;\;R_{iN}^{x_{FU}}\geq R_{2}, \end{array}\right.$$

where $R_{iN}^{x_{FU}}=\frac{1}{2}\log_{2}\left(1+\gamma_{iN,x_{FU}}^{}\right)$, $R_{2}$ is target rate for User FU.

**Proposition** **1.** *The outage probability of the User FU achieved by the NOMA scheme, assuming given antenna selection, can be computed as*

(15) $$\begin{array}{cc} P_{out,F}^{\left(I\right)}= & \sum_{k=0}^{K}\left(\begin{array}{c} K\\ k \end{array}\right){\left(-1\right)}^{k}e^{-\frac{k\gamma_{2}}{\Omega_{SF}\left(a_{1}-a_{2}\gamma_{2}\right)}}\times \\ & \left[\left(1-e^{-\frac{\gamma_{2}}{\Omega_{SN}\left(a_{1}-a_{2}\gamma_{2}\right)}}\right)+e^{-\frac{\gamma_{2}}{\Omega_{SN}\left(a_{1}-a_{2}\gamma_{2}\right)}}\left(1-e^{-\frac{\gamma_{2}}{\Omega_{NF}\rho }}\right)\right]. \end{array}$$

*where $a_{1}=\phi_{1}\rho$, $a_{2}=\phi_{2}\rho$.*

**Proof.**  See in Appendix A. □

### 3.2. Ergodic Capacity Analysis

We first define the ergodic capacity of User FU as below

(16) $$\begin{array}{c} C_{iF}=\frac{1}{2}\log_{2}\left(1+\gamma_{iF}\right)\\ \;\;\;\;\;\;\;\;\;\;\;\;=\frac{1}{2}\log_{2}\left(1+\frac{\phi_{1}\rho {\left|h_{iF}\right|}^{2}}{\phi_{2}\rho {\left|h_{iF}\right|}^{2}+1}\right)\\ \;\;\;\;\;\;\;\;\;\;\;\;=\frac{1}{2}\log_{2}\left(\frac{\phi_{2}\rho {\left|h_{iF}\right|}^{2}+\phi_{1}\rho {\left|h_{iF}\right|}^{2}+1}{\phi_{2}\rho {\left|h_{iF}\right|}^{2}+1}\right), \end{array}$$

Using $\phi_{1}=1-\phi_{2}$ we obtain new expression

(17) $$\begin{array}{c} C_{iF}=\frac{1}{2}\log_{2}\left(\frac{\phi_{2}\rho {\left|h_{iF}\right|}^{2}+\left(1-\phi_{2}\right)\rho {\left|h_{iF}\right|}^{2}+1}{\phi_{2}\rho {\left|h_{iF}\right|}^{2}+1}\right)\\ \;\;\;\;\;\;\;\;\;\;\;=\frac{1}{2}\log_{2}\left(\frac{\phi_{2}\rho {\left|h_{iF}\right|}^{2}+\rho {\left|h_{iF}\right|}^{2}-\phi_{2}\rho {\left|h_{iF}\right|}^{2}+1}{\phi_{2}\rho {\left|h_{iF}\right|}^{2}+1}\right)\\ \;\;\;\;\;\;\;\;\;\;\;=\frac{1}{2}\log_{2}\left(\frac{\rho {\left|h_{iF}\right|}^{2}+1}{\phi_{2}\rho {\left|h_{iF}\right|}^{2}+1}\right), \end{array}$$

Using $\mathrm{lo}g_{c}\left(x/y\right)=\;\mathrm{lo}g_{c}x-\;\mathrm{lo}g_{c}y$ it can be further obtained following formula

(18) $$C_{iF}=\frac{1}{2}\log_{2}\left(1+\rho {\left|h_{iF}\right|}^{2}\right)-\frac{1}{2}\log_{2}\left(1+\phi_{2}\rho {\left|h_{iF}\right|}^{2}\right),$$

(19) $$\begin{array}{c} C_{iF}^{\left(I\right)}=E\left\{C_{iF}\right\}\\ \;\;\;\;\;\;\;\;\;\;\;\;=\underset{I_{1}}{\underbrace{\frac{1}{2}\int_{0}^{\infty }\log_{2}\left(1+x\right)f_{X}\left(x\right)dx}}-\underset{I_{2}}{\underbrace{\frac{1}{2}\int_{0}^{\infty }\log_{2}\left(1+y\right)f_{Y}\left(y\right)dy}}, \end{array}$$

**Proposition** **2.** *The ergodic capacity of the User FU achieved by the NOMA scheme, assuming given antenna selection, can be computed as*

(20) $$C_{iF}^{\left(I\right)}=\frac{-e^{\frac{1}{\rho \Omega_{SF}}}}{2\ln2}\mathrm{Ei}\left(-\frac{1}{\rho \Omega_{SF}}\right)+\frac{e^{\frac{1}{\rho \phi_{2}\Omega_{SF}}}}{2\ln2}\mathrm{Ei}\left(-\frac{1}{\rho \phi_{2}\Omega_{SF}}\right).$$

**Proof.**  See in Appendix B. □

### 3.3. Consideration on Imperfect CSI

In this case, we consider the BS equipped two antennae. Due to imperfect CSI (ipCSI), the channel is measured with error component as below

(21) $$\begin{array}{cc} \begin{array}{cc} h_{q1}= & {\hat{h}}_{q1}+e_{q1} \end{array} & ,q1\in \left\{iN,iF\right\} \end{array}$$

The received signal can be rewritten at User NU and User FU as

(22) $$y_{q}=\left({\hat{h}}_{q}+\sigma_{e_{q}}\right)\left(\sqrt{\phi_{2}P_{S}}x_{NU}+\sqrt{\phi_{1}P_{S}}x_{FU}\right)+n_{q}$$

Then, the SINR to detect $x_{FU}$ in ipCSI case is given as

(23) $$\gamma_{iN,x_{FU}}^{ipCSI}=\frac{\phi_{1}\rho {\left|{\hat{h}}_{iN}\right|}^{2}}{\phi_{2}\rho {\left|{\hat{h}}_{iN}\right|}^{2}+\chi_{iN}},$$

where $\chi_{q}=\sigma_{e_{q}}^{2}\rho \left(\phi_{1}+\phi_{2}\right)+1$, $q\in \left\{iN,iF,NF\right\}$. After performing SIC, it can be obtained SINR to detect $x_{NU}$ as

(24) $$\gamma_{iN,x_{NU}}^{ipCSI}=\frac{\phi_{2}\rho {\left|{\hat{h}}_{iN}\right|}^{2}}{\chi_{iN}}$$

Similarly, SINR can be computed at User FU as

(25) $$\gamma_{iF}^{ipCSI}=\frac{\phi_{1}\rho {\left|{\hat{h}}_{iF}\right|}^{2}}{\phi_{2}\rho {\left|{\hat{h}}_{iF}\right|}^{2}+\chi_{iF}}$$

Considering on relay link NU-FU, the received signal can be achieved at User FU in this link as

(26) $$y_{NF}=\left({\hat{h}}_{NF}+\sigma_{e_{NF}}^{}\right)\sqrt{P_{N}}{\overset{⌢}{\;x}}_{FU}+n_{F}$$

Then, SNR can be calculated at User FU related to relay link as

(27) $$\gamma_{NF}^{ipCSI}=\frac{\rho {\left|h_{NF}\right|}^{2}}{\chi_{NF}}$$

In this step, outage performance is predicted that varying amount of performance happens due to impact of ipCSI term, it can be given as

(28) $$P_{out,F}^{(I),ipCSI}=\underset{{\bar{\Lambda }}_{1}}{\underbrace{\mathrm{Pr}\left(\gamma_{i^{*}N,x_{FU}}^{ipCSI}<\gamma_{2},\gamma_{i^{*}F}^{ipCSI}<\gamma_{2}\right)}}+\underset{{\bar{\Lambda }}_{2}}{\underbrace{\mathrm{Pr}\left(\gamma_{i^{*}N,x_{FU}}^{ipCSI}\geq \gamma_{2},\max\left(\gamma_{i^{*}F}^{ipCSI},\gamma_{NF}^{ipCSI}\right)<\gamma_{2}\right)}}.$$

Impact of ipCSI results in computations of two components ${\bar{\Lambda }}_{1},{\bar{\Lambda }}_{2}$ and they are given respectively as

(29) $$\begin{array}{ccc} {\bar{\Lambda }}_{1} & = & \mathrm{Pr}\left(\gamma_{i^{*}N,x_{FU}}^{ipCSI}<\gamma_{2},\gamma_{i^{*}F}^{ipCSI}<\gamma_{2}\right)\\ & = & \mathrm{Pr}\left({\left|{\hat{h}}_{i^{*}N}\right|}^{2}<\delta_{iN},{\left|{\hat{h}}_{i^{*}F}\right|}^{2}<\delta_{iF}\right)\\ & = & \int_{0}^{\delta_{iN}}f_{{\left|{\hat{h}}_{i^{*}N}\right|}^{2}}\left(x\right)\int_{0}^{\delta_{iF}}f_{{\left|{\hat{h}}_{i^{*}F}\right|}^{2}}\left(y\right)dxdy\\ & = & \sum_{a=1}^{K}\sum_{b=1}^{K}\left(\begin{array}{c} K\\ a \end{array}\right)\left(\begin{array}{c} K\\ b \end{array}\right)\frac{ab{\left(-1\right)}^{a+b-2}}{\Omega_{SN}\Omega_{SF}}\int_{0}^{\delta_{iN}}e^{-\frac{ax}{\Omega_{SN}}}\int_{0}^{\delta_{iF}}e^{-\frac{by}{\Omega_{SF}}}dxdy\\ & = & \sum_{a=1}^{K}\sum_{b=1}^{K}\left(\begin{array}{c} K\\ a \end{array}\right)\left(\begin{array}{c} K\\ b \end{array}\right){\left(-1\right)}^{a+b-2}\left(1-e^{-\frac{a\delta_{iN}}{\Omega_{SN}}}\right)\left(1-e^{-\frac{b\delta_{iF}}{\Omega_{SF}}}\right), \end{array}$$

where $\delta_{iN}=\frac{\gamma_{2}\chi_{iN}}{\rho \left(\phi_{1}-\phi_{2}\gamma_{2}\right)}$ and $\delta_{iF}=\frac{\gamma_{2}\chi_{iF}}{\rho \left(\phi_{1}-\gamma_{2}\phi_{2}\right)}$,

(30) $$\begin{array}{cc} {\bar{\Lambda }}_{2}= & \mathrm{Pr}\left(\gamma_{i^{*}N,x_{FU}}^{ipCSI}\geq \gamma_{2},\gamma_{i^{*}F}^{ipCSI}<\gamma_{2},\gamma_{NF}^{ipCSI}<\gamma_{2}\right)\\ = & \mathrm{Pr}\left({\left|{\hat{h}}_{i^{*}N}\right|}^{2}\geq \delta_{iN},{\left|{\hat{h}}_{i^{*}F}\right|}^{2}<\delta_{iF},{\left|h_{NF}\right|}^{2}<\delta_{NF}\right)\\ = & \sum_{a=1}^{K}\sum_{b=1}^{K}\left(\begin{array}{c} K\\ a \end{array}\right)\left(\begin{array}{c} K\\ b \end{array}\right)\frac{ab{\left(-1\right)}^{a+b-2}}{\Omega_{SN}\Omega_{SF}\Omega_{NF}}\int_{\delta_{iN}}^{\infty }e^{-\frac{ax}{\Omega_{SN}}}\int_{0}^{\delta_{iF}}e^{-\frac{by}{\Omega_{SF}}}\int_{0}^{\delta_{NF}}e^{-\frac{z}{\Omega_{NF}}}dxdydz\\ = & \sum_{a=1}^{K}\sum_{b=1}^{K}\left(\begin{array}{c} K\\ a \end{array}\right)\left(\begin{array}{c} K\\ b \end{array}\right){\left(-1\right)}^{a+b-2}e^{-\frac{a\delta_{iN}}{\Omega_{SN}}}\left(1-e^{-\frac{b\delta_{iF}}{\Omega_{SF}}}\right)\left(1-e^{-\frac{\delta_{NF}}{\Omega_{SF}}}\right), \end{array}$$

where $\delta_{NF}=\frac{\chi_{NF}\gamma_{2}}{\rho }$.

Substituting Equations (29) and (30) into Equation (28), $P_{out,F}^{(I),ipCSI}$ is formulated as

(31) $$P_{out,F}^{(I),ipCSI}=\sum_{a=1}^{K}\sum_{b=1}^{K}\left(\begin{array}{c} K\\ a \end{array}\right)\left(\begin{array}{c} K\\ b \end{array}\right){\left(-1\right)}^{a+b-2}\left(1-e^{-\frac{b\delta_{iF}}{\Omega_{SF}}}\right)\left[\left(1-e^{-\frac{a\delta_{iN}}{\Omega_{SN}}}\right)+e^{-\frac{a\delta_{iN}}{\Omega_{SN}}}\left(1-e^{-\frac{\delta_{NF}}{\Omega_{SF}}}\right)\right]$$

## 4. Scheme II: The Scenario of Two BSs Serving NOMA Users

This proposed TBS-based NOMA relaying network enhances the outage performance region by providing a downlink from two nearby BSs to simultaneous serve User NU, FU without requiring a complexity computation as antenna selection architecture in the previous section. In this scenario, two BSs transmit superimposed signals in downlink to the NU to perform signal processing at the NU due to low cost and complexity in system design. In this benchmark of TAS scheme, we compare TAS with a TBS to provide guidelines for practical design of NOMA scheme with improved outage performance. According to NOMA protocol, optimal power allocation factors including $\left(\phi_{1}\right)$, $\left(\phi_{2}\right)$ need to be further considered to satisfy fairness among User FU and User NU. As expected, each signal in ($x_{FU}$, $x_{NU}$) is transmitted by two sources, then half power is allocated for each. This characterization requires information feedback to two sources of simultaneous transmitting, and this will be investigated in future work as it is beyond of the scope of our current paper.

The received signal can be obtained at the User NU as below

(32) $$\begin{array}{c} y_{SN}=h_{S1N}\left(\sqrt{\frac{P_{S}\phi_{1}}{2}}x_{FU}+\sqrt{\frac{P_{S}\phi_{2}}{2}}x_{NU}\right)\\ +h_{S2N}\left(\sqrt{\frac{P_{S}\phi_{1}}{2}}x_{FU}+\sqrt{\frac{P_{S}\phi_{2}}{2}}x_{NU}\right)+n_{SN}. \end{array}$$

Similarly, the received signal at the User FU can be computed by

(33) $$\begin{array}{c} y_{SF}=h_{S1F}\left(\sqrt{\frac{P_{S}\phi_{1}}{2}}x_{FU}+\sqrt{\frac{P_{S}\phi_{2}}{2}}x_{NU}\right)\\ +h_{S2F}\left(\sqrt{\frac{P_{S}\phi_{1}}{2}}x_{FU}+\sqrt{\frac{P_{S}\phi_{2}}{2}}x_{NU}\right)+n_{SF}. \end{array}$$

We assume that the two signals from two BS transmit to User FU in direct link in same time thanks to the required perfect synchronization operation required. It is assumed that FU can be able to detect its signal in the direct link while the NU first detects the FU’s signal and then further detects its own signal. Therefore, the received SINR at FU to detect information $x_{FU}$ is expressed as

(34) $$\gamma_{SF}=\frac{\frac{\phi_{1}}{2}\sum_{i=0}^{1}{\left|h_{SiF}\right|}^{2}}{\frac{\phi_{2}}{2}\sum_{i=0}^{1}{\left|h_{SiF}\right|}^{2}+\frac{1}{\rho }}=\frac{\frac{\rho \;\phi_{1}}{2}\sum_{i=0}^{1}{\left|h_{SiF}\right|}^{2}}{\frac{\rho \;\phi_{2}}{2}\sum_{i=0}^{1}{\left|h_{SiF}\right|}^{2}+1}.$$

On the other hand, User NU performs SIC to decode symbols $x_{NU}$ because of better channel quality as observation in link BS-NU compared with link BS-FU. In particular, the received SINR at User NU to detect User FU’s signal before further signal processing is written as

(35) $$\gamma_{SF\to SN}=\frac{\frac{\rho \;\phi_{1}}{2}\sum_{i=0}^{1}{\left|h_{SiN}\right|}^{2}}{\frac{\rho \;\phi_{2}}{2}\sum_{i=0}^{1}{\left|h_{SiN}\right|}^{2}+1}.$$

Then, the received SINR at User NU to identify own signal can be formulated by

(36) $$\gamma_{SN}=\frac{\rho \;\phi_{2}}{2}\sum_{i=0}^{1}{\left|h_{SiN}\right|}^{2}.$$

During the second phase, only User NU equipping the transmit power of $P_{N}$ retransmits the decoded symbols and to User FU at far distance.

(37) $$\gamma_{NF}=\rho {\left|h_{NF}\right|}^{2}.$$

### 4.1. Outage Probability Analysis

In this section, we present the exact closed-form solution for outage probability over the two simultaneous transmit antenna at the BS with its required target rate. If both the direct link BS-FU and relaying link BS-NU-FU are unable to satisfy the predefined target data rate, outage behavior can be extracted from the proposed system. This study only focuses on outage performance of User FU and it is given by

(38) $$\begin{array}{c} P_{out}^{\left(II\right)}=\mathrm{Pr}\left\{\min\left(\gamma_{SN},\gamma_{NF}\right)<\gamma_{2}\right\}\mathrm{Pr}\left\{\gamma_{SF}<\gamma_{2}\right\}\mathrm{Pr}\left\{\gamma_{SF\to SN}>\gamma_{2}\right\}\\ \;\;\;\;\;\;\;\;\;\;\;\;\;\;\;+\mathrm{Pr}\left\{\gamma_{SF}<\gamma_{2}\right\}\times \mathrm{Pr}\left\{\gamma_{SF\to SN}<\gamma_{2}\right\}, \end{array},$$

**Proposition** **3.** *The outage probability in TBS-based NOMA mode can be computed as*

(39) $$\begin{array}{cc} P_{out}^{\left(II\right)}= & \left[1-\exp\left(-\frac{2\gamma_{2}}{\phi_{2}\rho \Omega_{SN}}-\frac{\gamma_{2}}{\rho \Omega_{NF}}\right)\right]\left(1+\frac{2\gamma_{2}}{\phi_{2}\rho \Omega_{SN}}\right)\\ \times & \;\left[1-\exp\left(-\frac{\Gamma_{1}}{\Omega_{SF}}\right)\left(1+\frac{\Gamma_{1}}{\Omega_{SF}}\right)\right]\times \exp\left(-\frac{\Gamma_{1}}{\Omega_{SN}}\right)\left(1+\frac{\Gamma_{1}}{\Omega_{SN}}\right)\\ + & \left[1-\exp\left(-\frac{\Gamma_{1}}{\Omega_{SF}}\right)\left(1+\frac{\Gamma_{1}}{\Omega_{SF}}\right)\right]\times \left[1-\exp\left(-\frac{\Gamma_{1}}{\Omega_{SN}}\right)\left(1+\frac{\Gamma_{1}}{\Omega_{SN}}\right)\right] \end{array}$$

*where $\Gamma_{1}=\frac{2\gamma_{2}}{\rho \left\{\phi_{1}-\phi_{2}\gamma_{2}\right\}}$, $\gamma_{2}=2^{2R_{2}}-1$, $R_{2}$ is target rate for User FU.*

**Proof.**  We continue compute the components in expression of outage event as follow

(40) $$\begin{array}{cc} \mathrm{Pr}\left\{\min\left(\gamma_{SN},\gamma_{NF}\right)<\gamma_{2}\right\} & =1-\exp\left(-\frac{2\gamma_{2}}{\phi_{2}\rho \Omega_{SN}}\right)\sum_{i=0}^{1}{\left(-\frac{2\gamma_{2}}{\phi_{2}\rho \Omega_{SN}}\right)}^{i}\frac{1}{i!}\exp\left(-\frac{\gamma_{2}}{\rho \Omega_{NF}}\right)\\ & =1-\exp\left(-\frac{2\gamma_{2}}{\phi_{2}\rho \Omega_{SN}}-\frac{\gamma_{2}}{\rho \Omega_{NF}}\right)\left(1+\frac{2\gamma_{2}}{\phi_{2}\rho \Omega_{SN}}\right). \end{array}$$

(41) $$\begin{array}{cc} \mathrm{Pr}\left\{\gamma_{SF}<\gamma_{2}\right\} & =1-\exp\left(-\frac{\Gamma_{1}}{\Omega_{SF}}\right)\sum_{i=0}^{1}{\left(-\frac{\Gamma_{1}}{\Omega_{SF}}\right)}^{i}\frac{1}{i!}\\ & =1-\exp\left(-\frac{\Gamma_{1}}{\Omega_{SF}}\right)\left(1+\frac{\Gamma_{1}}{\Omega_{SF}}\right). \end{array}$$

and

(42) $$\begin{array}{cc} \mathrm{Pr}\left\{\gamma_{SF\to SN}>\gamma_{2}\right\} & =\exp\left(-\frac{\Gamma_{1}}{\Omega_{SN}}\right)\sum_{i=0}^{1}{\left(-\frac{\Gamma_{1}}{\Omega_{SN}}\right)}^{i}\frac{1}{i!}\\ & =\exp\left(-\frac{\Gamma_{1}}{\Omega_{SN}}\right)\left(1+\frac{\Gamma_{1}}{\Omega_{SN}}\right). \end{array}$$

where $\Gamma_{1}=\frac{2\gamma_{2}}{\rho \left\{\phi_{1}-\phi_{2}\gamma_{2}\right\}},\;\;and\;\frac{\gamma_{2}}{\gamma_{2}+1}<\phi_{1}<1$. Plugging Equations (40)–(42) into Equation (38) the proof is complete proved. □

### 4.2. Ergodic Capacity in TBS-Based NOMA

In this section, we focus on the ergodic capacity analysis of the proposed TBS-based NOMA scheme. By without considering any delay constraints, the ergodic capacity can be quantified as the long-term average data rate that can be attained.

(43) $$\begin{array}{cc} C_{SiF} & =\frac{1}{2}\log_{2}\left(1+\gamma_{SF}\right)\\ & =\frac{1}{2}\log_{2}\left(1+\frac{\frac{\phi_{1}\rho }{2}\sum_{i=0}^{1}{\left|h_{SiF}\right|}^{2}}{\frac{\phi_{2}\rho }{2}\sum_{i=0}^{1}{\left|h_{SiF}\right|}^{2}+1}\right)\\ & =\frac{1}{2}\log_{2}\left(\frac{\frac{\phi_{2}\rho }{2}{\left|h_{SiF}\right|}^{2}+\frac{\phi_{1}\rho }{2}\sum_{i=0}^{1}{\left|h_{SiF}\right|}^{2}+1}{\frac{\phi_{2}\rho }{2}\sum_{i=0}^{1}{\left|h_{SiF}\right|}^{2}+1}\right), \end{array}$$

By using such a constraint, it can be further analyzed as

(44) $$\begin{array}{cc} C_{SiF} & =\frac{1}{2}\log_{2}\left(\frac{\frac{\phi_{2}\rho }{2}\sum_{i=0}^{1}{\left|h_{SiF}\right|}^{2}+\left(1-\phi_{2}\right)\frac{\rho }{2}\sum_{i=0}^{1}{\left|h_{SiF}\right|}^{2}+1}{\frac{\phi_{2}\rho }{2}{\left|h_{SiF}\right|}^{2}+1}\right)\\ & =\frac{1}{2}\log_{2}\left(\frac{\frac{\phi_{2}\rho }{2}\sum_{i=0}^{1}{\left|h_{SiF}\right|}^{2}+\frac{\rho }{2}\sum_{i=0}^{1}{\left|h_{SiF}\right|}^{2}-\frac{\phi_{2}\rho }{2}\sum_{i=0}^{1}{\left|h_{SiF}\right|}^{2}+1}{\frac{\phi_{2}\rho }{2}\sum_{i=0}^{1}{\left|h_{SiF}\right|}^{2}+1}\right)\\ & =\frac{1}{2}\log_{2}\left(\frac{\frac{\rho }{2}\sum_{i=0}^{1}{\left|h_{SiF}\right|}^{2}+1}{\frac{\phi_{2}\rho }{2}\sum_{i=0}^{1}{\left|h_{SiF}\right|}^{2}+1}\right), \end{array}$$

It is noted that $\mathrm{lo}g_{w}\left(u/v\right)=\;\mathrm{lo}g_{w}u-\;\mathrm{lo}g_{w}v$, the ergodic capacity in such case can be rewritten as

(45) $$C_{SiF}=\frac{1}{2}\log_{2}\left(1+\frac{\rho }{2}\sum_{i=0}^{1}{\left|h_{SiF}\right|}^{2}\right)-\frac{1}{2}\log_{2}\left(1+\frac{\phi_{2}\rho }{2}\sum_{i=0}^{1}{\left|h_{SiF}\right|}^{2}\right)$$

**Proposition** **4.** *Ergodic capacity in TBS-based NOMA can be formulated as*

(46) $$\begin{array}{cc} C_{SiF}^{\left(II\right)} & =\frac{1}{2\ln(2)}\left\{ae^{a}\mathrm{Ei}\left(-a\right)-e^{a}\mathrm{Ei}\left(-a\right)+1-\left(be^{b}\mathrm{Ei}\left(-b\right)\left(b-1\right)+1\right)\right\}\\ & =\frac{1}{2\ln(2)}\left\{ae^{a}\mathrm{Ei}\left(-a\right)-e^{a}\mathrm{Ei}\left(-a\right)-be^{b}\mathrm{Ei}\left(-b\right)\left(b-1\right)\right\}. \end{array}$$

*where $a=\frac{2}{\rho \Omega_{SF}}$, $b=\frac{2}{\phi_{2}\rho \Omega_{SF}}$.*

**Proof.**  *Proof*: See in Appendix C. □

### 4.3. Imperfect CSI Evaluation in Scheme II

Similarly, due to ipSIC happens at Scheme II, $P_{out}^{(II),ipCSI}$ is rewritten as

(47) $$\begin{array}{cc} P_{out}^{(II),ipCSI}= & \mathrm{Pr}\left\{\min\left(\gamma_{SN}^{ipCSI},\gamma_{NF}^{ipCSI}\right)<\gamma_{2}\right\}\mathrm{Pr}\left\{\gamma_{SF}^{ipCSI}<\gamma_{2}\right\}\mathrm{Pr}\left\{\gamma_{SF\to SN}^{ipCSI}>\gamma_{2}\right\}\\ + & \mathrm{Pr}\left\{\gamma_{SF}^{ipCSI}<\gamma_{2}\right\}\times \mathrm{Pr}\left\{\gamma_{SF\to SN}^{ipCSI}<\gamma_{2}\right\}, \end{array}$$

where $\gamma_{SN}^{ipCSI}=\frac{\frac{\rho \;\;\phi_{2}}{2}\sum_{i=0}^{1}{\left|h_{SiN}\right|}^{2}}{\frac{\rho }{2}\;\;\chi_{SN}+1}$, $\gamma_{NF}^{ipCSI}=\frac{\rho {\left|h_{NF}\right|}^{2}}{\rho \;\;\chi_{NF}+1}$, $\gamma_{SF}^{ipCSI}=\frac{\frac{\rho \;\phi_{1}}{2}\sum_{i=0}^{1}{\left|h_{SiF}\right|}^{2}}{\frac{\rho \;\phi_{2}}{2}\sum_{i=0}^{1}{\left|h_{SiF}\right|}^{2}+\frac{\rho }{2}\;\;\chi_{SF}+1}$ and $\gamma_{SF\to SN}^{ipCSI}=\frac{\frac{\rho \;\phi_{1}}{2}\sum_{i=0}^{1}{\left|h_{SiN}\right|}^{2}}{\frac{\rho \;\phi_{2}}{2}\sum_{i=0}^{1}{\left|h_{SiN}\right|}^{2}+\frac{\rho }{2}\;\;\chi_{SN}+1}$.

By applying solving method of Equation (39), Equation (47) is then solved as follow

(48) $$\begin{array}{cc} P_{out}^{(II),ipCSI}= & \left[1-\exp\left(-\frac{2\gamma_{2}}{\rho \;\;\phi_{2}\Omega_{SN}}\left(\frac{\rho }{2}\;\;\chi_{SN}+1\right)-\frac{\gamma_{2}\left(\rho \;\;\chi_{NF}+1\right)}{\rho }\right)\left(1+\frac{2\gamma_{2}}{\rho \;\;\phi_{2}\Omega_{SN}}\left(\frac{\rho }{2}\;\;\chi_{SN}+1\right)\right)\right]\\ \times & \left[1-\exp\left(-\frac{2\gamma_{2}}{\rho \Omega_{SF}\;\;\left(\phi_{1}-\phi_{2}\gamma_{2}\right)}\left(\frac{\rho }{2}\;\;\chi_{SN}+1\right)\right)\left(1+\frac{2\gamma_{2}}{\rho \Omega_{SF}\;\;\left(\phi_{1}-\phi_{2}\gamma_{2}\right)}\left(\frac{\rho }{2}\;\;\chi_{SN}+1\right)\right)\right]\\ \times & \exp\left(-\frac{2\gamma_{2}}{\rho \Omega_{SN}\;\;\left(\phi_{1}-\phi_{2}\gamma_{2}\right)}\left(\frac{\rho }{2}\;\;\chi_{SN}+1\right)\right)\left(1+\frac{2\gamma_{2}}{\rho \Omega_{SN}\;\;\left(\phi_{1}-\phi_{2}\gamma_{2}\right)}\left(\frac{\rho }{2}\;\;\chi_{SN}+1\right)\right)\\ + & \left[1-\exp\left(-\frac{2\gamma_{2}}{\rho \Omega_{SF}\;\;\left(\phi_{1}-\phi_{2}\gamma_{2}\right)}\left(\frac{\rho }{2}\;\;\chi_{SN}+1\right)\right)\left(1+\frac{2\gamma_{2}}{\rho \Omega_{SF}\;\;\left(\phi_{1}-\phi_{2}\gamma_{2}\right)}\left(\frac{\rho }{2}\;\;\chi_{SN}+1\right)\right)\right]\\ \times & \left[1-\exp\left(-\frac{2\gamma_{2}}{\rho \Omega_{SN}\;\;\left(\phi_{1}-\phi_{2}\gamma_{2}\right)}\left(\frac{\rho }{2}\;\;\chi_{SN}+1\right)\right)\left(1+\frac{2\gamma_{2}}{\rho \Omega_{SN}\;\;\left(\phi_{1}-\phi_{2}\gamma_{2}\right)}\left(\frac{\rho }{2}\;\;\chi_{SN}+1\right)\right)\right] \end{array}$$

**Remark** **1.** *Due to half power allocated to the BSs in Scheme II, it is predicted that outage performance in Scheme II is worse than that in Scheme I. However, two BSs required to serve two NOMA users is interesting application since it is deployed in current cellular network. Single antenna is usually equipped in the BS and mobile user in existing networks. Furthermore, a performance comparison between Scheme I and Scheme II should be considered.*

## 5. Scheme III: Multi-User NOMA

In this situation, $h_{sd_{i}},i=1,2,...,M$ are denoted as channel from the BS to *M* NOMA users. Two main groups of users can be divided based on their locations. Without loss of generality, the channel gains of M users are sorted as ${\left|h_{sd_{1}}\right|}^{2}\leq {\left|h_{sd_{2}}\right|}^{2}\leq \ldots \leq {\left|h_{sd_{M}}\right|}^{2}$.

**Figure 3:**
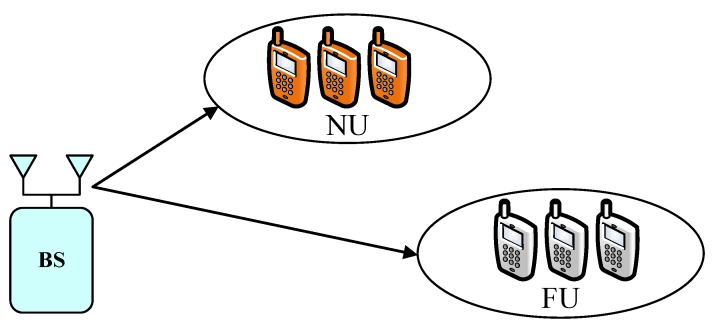
System model for Multi-User NOMA network.

In a multi-user scenario illustrated in in Figure 3, the m-th user requires SIC to detect and cancel the k-th user information $\left(k\leq m\right)$. Then, the m-th user detects and decodes its own signals as procedure of NOMA scheme. If the m-th user cannot detect the k-th user information, outage behavior is considered. Therefore, the m-th user meets outage probability and it can be formulated as [36,37]

(49) $$P_{out,m}^{\left(III\right)}=\mathrm{Pr}\left({\left|h_{m}\right|}^{2}<\varepsilon_{m}^{*}\right),$$

where $\varepsilon_{m}^{*}=\max\left(\varepsilon_{1},\varepsilon_{2},\ldots ,\varepsilon_{m}\right)$, $\varepsilon_{k}=\frac{\gamma_{k}}{\rho \left(\phi_{k}-\gamma_{k}\sum_{j=k+1}^{M}\phi_{j}\right)}$ for k<M, $\gamma_{k}=2^{R_{k}}-1$. It is denoted that $R_{k}$ as the target rate at k-th user. In particular, $\phi_{k}>\gamma_{k}\sum_{j=k+1}^{M}\phi_{j}$ is required condition to existence of Equation (49).

By exploiting statistics and binomial theorem [38], the PDF and CDF of the m-th users channel gain ${\left|h_{m}\right|}^{2}$ can be expressed as

(50) $$\begin{array}{cc} f_{{\left|h_{m}\right|}^{2}}\left(x\right)= & \frac{M!}{\left(m-1\right)!\left(M-m\right)!}f_{{\left|h\right|}^{2}}\left(x\right)\\ \; & \times {\left(F_{{\left|h\right|}^{2}}\left(x\right)\right)}^{m-1}{\left(1-F_{{\left|h\right|}^{2}}\left(x\right)\right)}^{M-m}, \end{array}$$

and

(51) $$\begin{array}{cc} F_{{\left|h_{m}\right|}^{2}}\left(x\right)= & \frac{M!}{\left(m-1\right)!\left(M-m\right)!}\sum_{i=0}^{M-m}\left(\begin{array}{c} M-m\\ i \end{array}\right)\\ \; & \times \frac{{\left(-1\right)}^{i}}{m+i}{\left(F_{{\left|h\right|}^{2}}\left(x\right)\right)}^{m+i}. \end{array}$$

Finally, the outage performance of the m-th user can be evaluated by

(52) $$\begin{array}{cc} P_{out,m}^{\left(III\right)}= & \mathrm{Pr}\left({\left|h_{m}\right|}^{2}<\varepsilon_{m}^{*}\right)\\ = & \Theta_{m}\sum_{k=0}^{M-m}\left(\begin{array}{c} M-m\\ k \end{array}\right)\frac{{\left(-1\right)}^{k}}{m+k}{\left(1-e^{-\frac{\varepsilon_{m}^{*}}{\Omega_{h}}}\right)}^{K\left(m+k\right)}\\ = & \Theta_{m}\sum_{k=0}^{M-m}\sum_{r=0}^{K\left(m+k\right)}\left(\begin{array}{c} M-m\\ k \end{array}\right)\left(\begin{array}{c} K\left(m+k\right)\\ r \end{array}\right)\frac{{\left(-1\right)}^{m+K\left(m+k\right)-r}}{m+k}e^{-\frac{\varepsilon_{m}^{*}\left(K\left(m+k\right)-r\right)}{\Omega_{h}}}, \end{array}$$

where $\Theta_{m}=\frac{M!}{\left(m-1\right)!\left(M-m\right)!}$.

## 6. Numerical Results

In this section, numerical examples are presented to validate the outage performance of the downlink NOMA network under Rayleigh fading channels with two transmission policies. Moreover, system performance of NOMA is compared system performance in terms of outage and ergodic capacity performance with different parameters in such networks, where antenna transmit architecture is scheduled from derived expressions. The channel gains are set $\Omega_{SN}^{}=d_{SN}^{-\mu }=1$, $\Omega_{NF}^{}=d_{NF}^{-\mu }=1$, $\Omega_{SF}^{}=d_{SF}^{-\mu }=1$. Power allocation factors are $\phi_{1}=0.8$, $\phi_{2}=0.2$ except for specific cases. Without loss of generality, we assume the distance in pair of nodes is normalized to unity. We set $\sigma_{e}^{2}=\sigma_{e_{iN}}^{2}=\sigma_{e_{iF}}^{2}=\sigma_{e_{NF}}^{2}$ denotes the channel estimation error.

**Figure 4:**
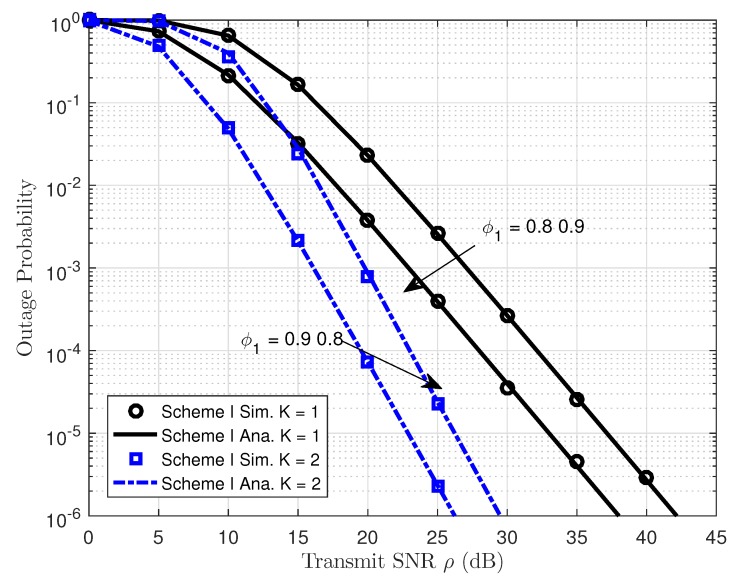
Outage Probability versus transmit SNR in Scheme I.

In Figure 4, the outage probability performance versus system SNR for User FU as employing different power allocation parameters can be clearly observed. First, the exact analytical results and simulation results are in strict agreement. Secondly, higher power allocation factor dedicated for User FU leads to better outage probability shown in entire SNR regimes. The reason for this is that transmitting SNR at the BS term contributes significantly to the outage behavior from the viewpoint of mathematical analysis. Moreover, as the system SNR increases, the outage probability decreases. Another important observation is that the outage probability for User FU of NOMA with two transmitting antennae at the BS outperforms the BS with single antenna.

**Figure 5:**
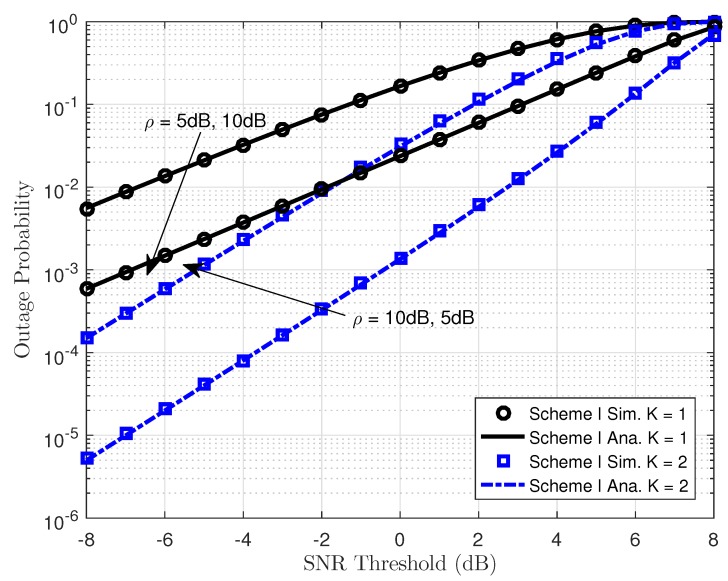
Outage probability as a function of the SNR threshold in Scheme I.

**Figure 6:**
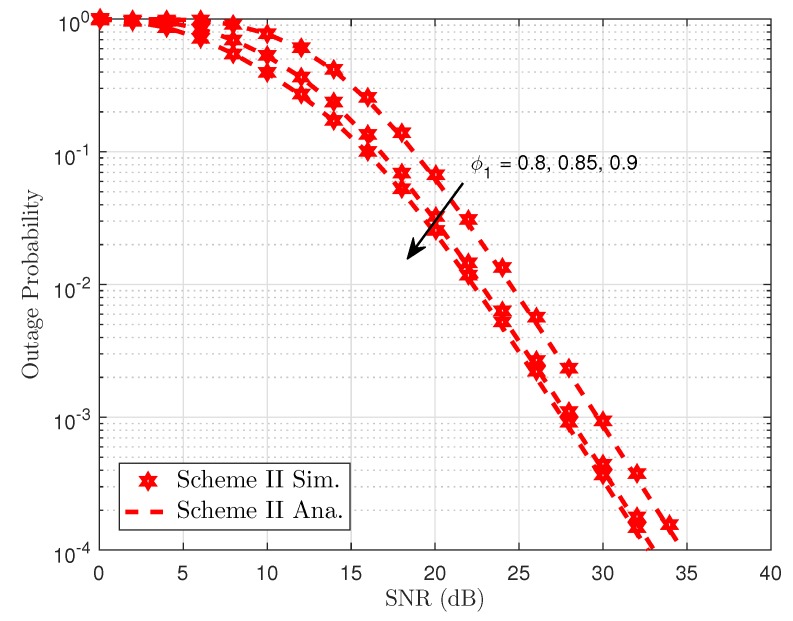
Outage Probability versus transmit SNR in Scheme II.

**Figure 7:**
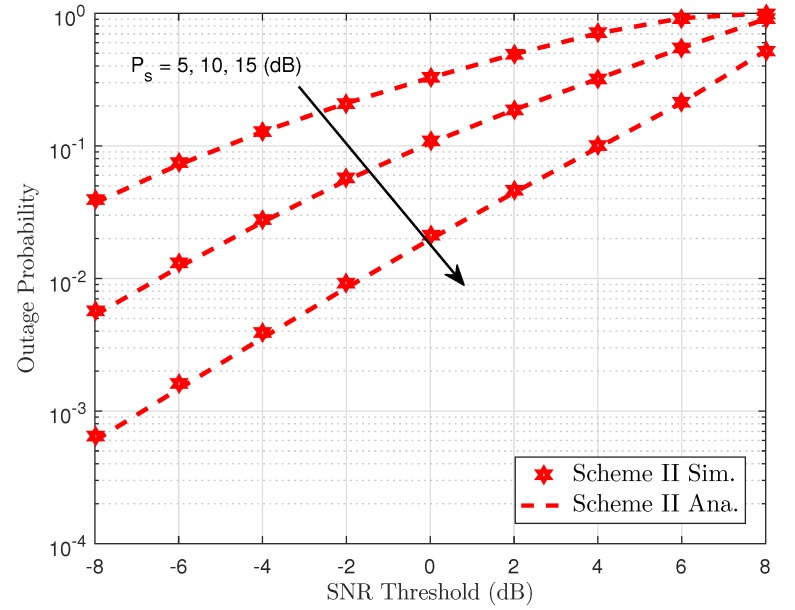
Outage probability as a function of SNR threshold in Scheme II.

Figure 5 shows the outage performance against the transmit SNR of the proposed NOMA scheme with the varying transmit power of the BS. We can see from the simulation results that outage can be accepted with very low SNR threshold level and outage event occurred at SNR threshold is 8 dB, which indicates that although selected antenna provides improved performance, the required higher SNR threshold makes the system worse. The main reason is explained as the transmit SNR ρ make the outage behavior change its performance. Greater antennae at the BS help improve the performance of destinations as higher diversity is achieved. It can achieve a similar result compared to the Scheme I, which considers the effect of the same parameters in the outage performance. Thus, in the following simulations, we only consider the concerned outage event in Scheme II. Power allocation factors and transmit power at the BS contribute to varying outage performance of User FU. Such results can be seen in Figure 6 and Figure 7.

**Figure 8:**
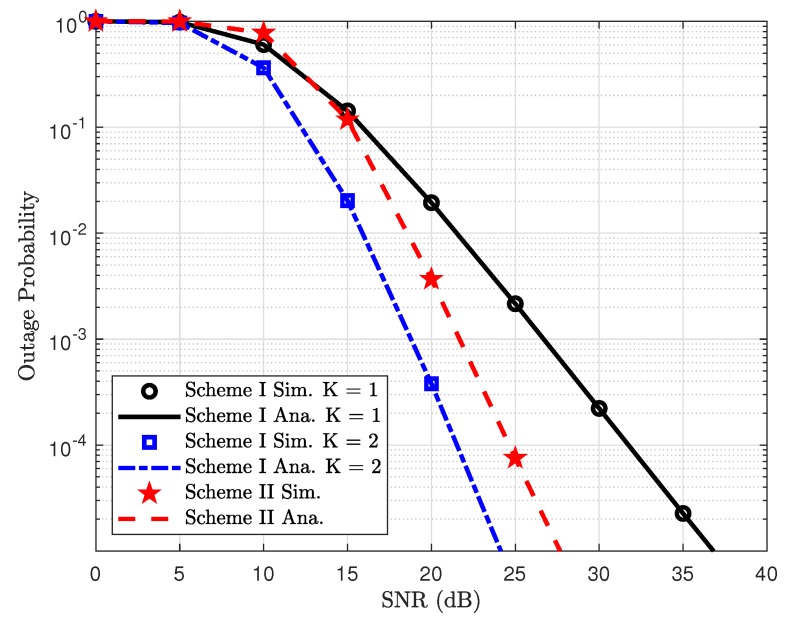
Comparison study on outage probability between Scheme I and Scheme II.

Figure 8 compares outage performance between Scheme I and Scheme II. In particular, the proposed Scheme I provides better outage performance, which benefits from the antenna selection architecture to serve multiple users in such NOMA system. It is confirmed that more chances to select better signal as K=2 and this case exhibits better performance.

**Figure 9:**
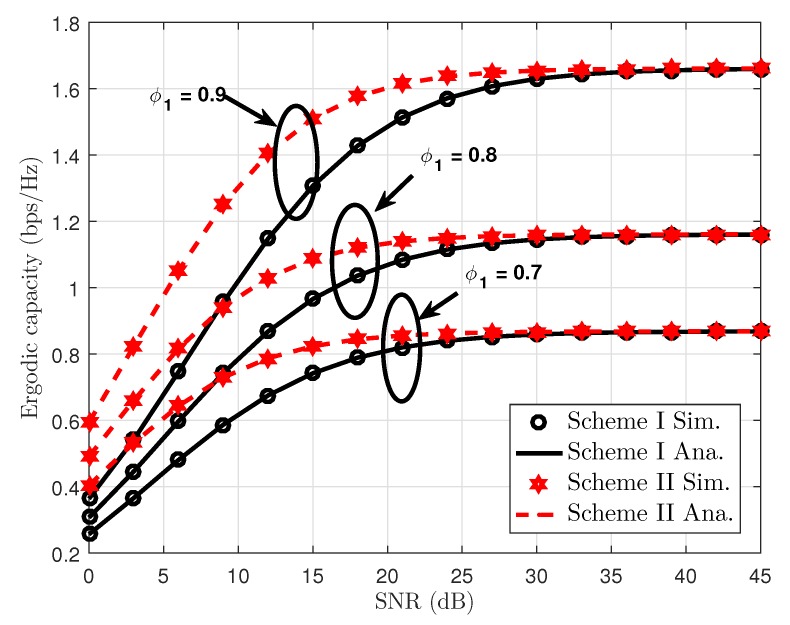
Ergodic capacity performance versus SNR at Scheme I and Scheme II.

Figure 9 depicts the ergodic capacity performance of our proposed scheme as varying power allocation coefficients. From this figure, the ergodic rate of User FU increases, and the performance gap related to ergodic rates of User FU remains at a stable level at high SNR. This is because ergodic capacity is an increasing function of SNR threshold from the theoretical analysis. Results reveal that our proposal has an obvious advantage over the Scheme II, with better ergodic capacity compared with Scheme I. Furthermore, there is a gap between Scheme I and Scheme II at low SNR, but the same performance can be seen in two schemes at SNR higher than 30dB. One possible reason is that when User FU is limited by high SNR threshold and then corresponding high SNR, the amount of increased ergodic capacity can be seen clearly as varying small amount of power allocation factor.

**Figure 10:**
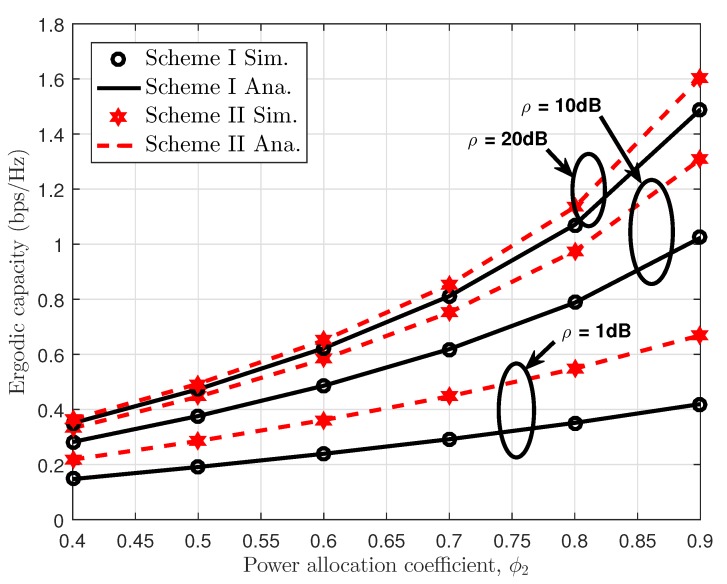
Ergodic capacity comparison with respect to the power allocation coefficient $\phi_{2}$ in Scheme I and Scheme II.

**Figure 11:**
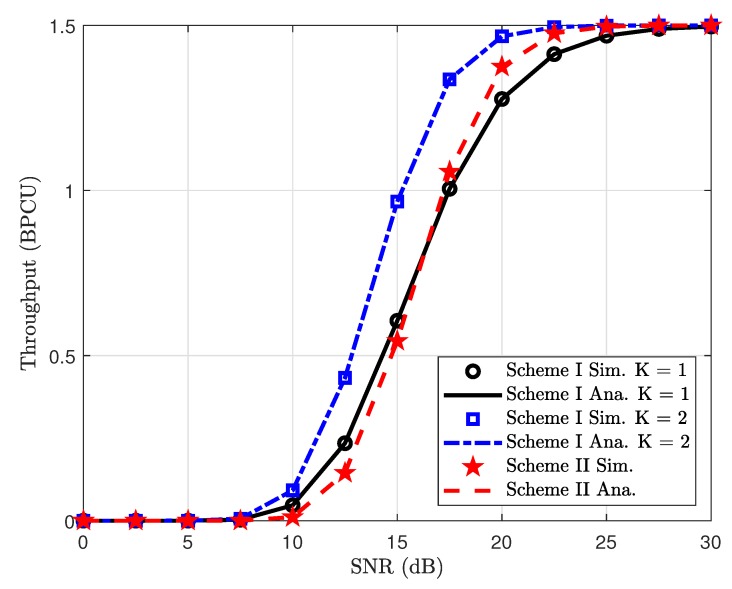
Throughput comparison between Scheme I and Scheme II, with $\phi_{1}=0.9$ and $\phi_{2}=0.9$.

In Figure 10, we numerically investigate the impact of power allocation coefficients on the ergodic capacity performance of two transmission policies in such NOMA system. It can be seen clearly that higher transmit power at the BS and higher power allocation factors result in enhanced ergodic capacity performance. On the other hand, Figure 11 examines the throughput metric for Scheme I and Scheme II. In this figure, performance gaps among these considered cases exist in the middle range from SNR = 10 (dB) to SNR = 25 (dB). At very low regime and high regime of SNR, such throughput is seen as the same performance for all cases.

**Figure 12:**
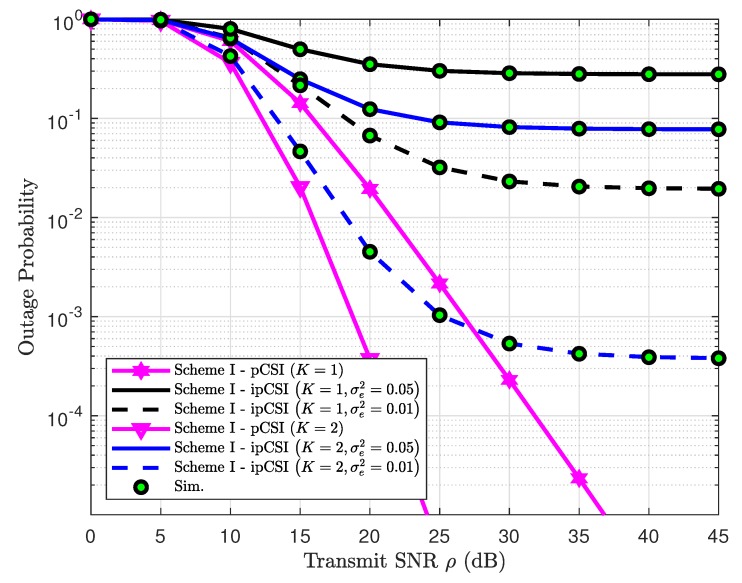
Impact of ipCSI on outage performance in Scheme I.

**Figure 13:**
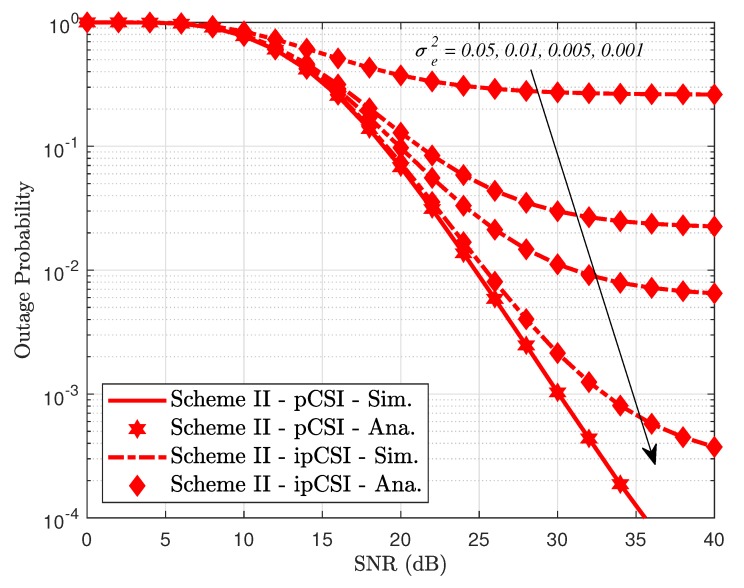
Impact of ipCSI on outage performance in Scheme II.

In Figure 12, we evaluate outage performance of the proposed system in Scheme I where it exhibits better performance as two antennae are equipped at the BS. Unfortunately, the higher level of channel estimation error makes the system performance its worst case. However, such outage performance remains stable at high SNR regardless of level of channel estimation imperfection. On the other hand, the outage behavior improves significantly in pCSI case at high SNR. A similar trend can be observed at Figure 13 for Scheme II in which a higher level of channel estimation imperfection results in worse outage behavior.

**Figure 14:**
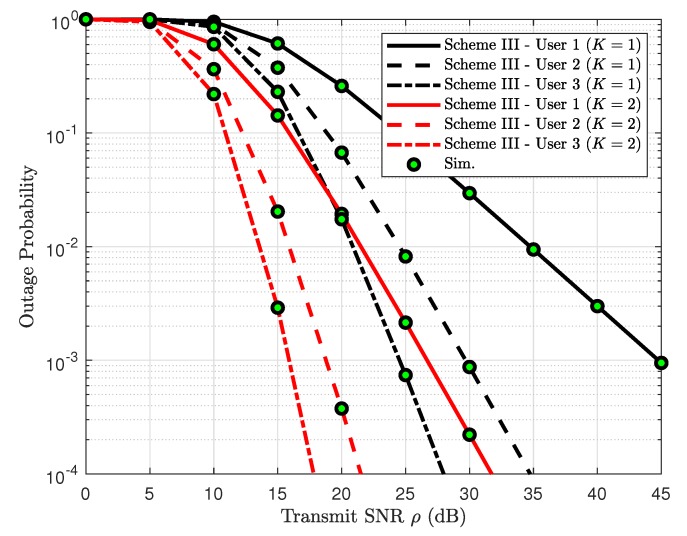
Consideration of scenario of multiple users in Scheme III, with M=3, $\phi_{1}=0.5$, $\phi_{2}=0.4$, $\phi_{3}=0.1$, $R_{1}=0.2$, $R_{2}=1$ and $R_{3}=2$.

Figure 14 plots the outage probability of three users versus SNR with implementation of two-antenna BS. It can be further confirmed that the exact outage probability lines match tightly with the Monte Carlo simulation results. The different power allocation coefficients assigned to each user is the main reason for exhibiting dissimilar outage performance. Another observation is that when several users’ QoS are met, outage performance still exhibits reasonable value.

## 7. Conclusions

This paper first considered real scenarios regarding NOMA users who are being served by two-antenna BS (Scheme I) or two single-antenna BS (Scheme II). Considering that the number of antennae has an impact on the gained SNR computation at destination, we have theoretically derived the outage probabilities of the far User FU. In Scheme II, two nearby BSs transmit a superimposed signal to the User NU to forward the signal to far User FU. Simulation results demonstrated that Scheme I has better outage probability compared with the others. In contrast, the ergodic capacity in Scheme II exhibited improved performance in comparison with that in Scheme I at several scenarios in the NOMA relaying system, and then helped choose which one was suitable for real implementation. Scheme III introduced the scenario of existence of multiple users in practice. It can be seen with each user having different channel condition results in different outage performance. In addition, with the metric of outage probability, we further evaluated ergodic capacity of such NOMA and theoretically derived ergodic performance, and two considered policies are examined. It is found that no matter which transmission policy is selected, the power allocation coefficients and transmit power at the BS are the main parameters affecting system performance in terms of outage behavior and ergodic capacity. These systems are suitable to implement wireless sensor networks where high demand of massive connections from sensors are satisfied. In future work, full multiple-antenna systems will be studied to provide further comparison related to these considered systems.

## Appendix A

**Proof** **of Proposition 1**. The outage probability can be expressed as

(A1) $$P_{out,F}^{\left(I\right)}=\underset{\Omega_{1}}{\underbrace{\mathrm{Pr}\left(\gamma_{i^{*}N}^{x_{F}}<\gamma_{2},\gamma_{i^{*}F}<\gamma_{2}\right)}}+\underset{\Omega_{2}}{\underbrace{\mathrm{Pr}\left(\gamma_{i^{*}N}^{x_{F}}\geq \gamma_{2},\max\left(\gamma_{i^{*}F},\gamma_{NF}\right)<\gamma_{2}\right)}},$$

Therefore, the outage probability of the User FU can be further expressed as

(A2) $$\Omega_{1}=\mathrm{Pr}\left(\frac{a_{1}{\left|h_{i^{*}N}\right|}^{2}}{a_{2}{\left|h_{i^{*}N}\right|}^{2}+1}<\gamma_{2},\frac{b_{1}{\left|h_{i^{*}F}\right|}^{2}}{b_{2}{\left|h_{i^{*}F}\right|}^{2}+1}<\gamma_{2}\right)$$

(A3) $$\Omega_{1}=\underset{\Omega_{1A}}{\underbrace{\mathrm{Pr}\left({\left|h_{i^{*}N}\right|}^{2}<\frac{\gamma_{2}}{a_{1}-a_{2}\gamma_{2}}\right)}}\underset{\Omega_{1B}}{\underbrace{\mathrm{Pr}\left({\left|h_{i^{*}F}\right|}^{2}<\frac{\gamma_{2}}{b_{1}-b_{2}\gamma_{2}}\right)}}.$$

It is noted that CDF of channel corresponding the selected antenna at the BS can be shown as

(A4) $$\begin{array}{cc} F_{{\left|h_{i^{*}N}\right|}^{2}}\left(x\right) & =\mathrm{Pr}\left({\left|h_{i^{*}N}\right|}^{2}<x\right)\\ & =\sum_{i=1}^{K}\mathrm{Pr}(i^{*}=i)\mathrm{Pr}\left({\left|h_{iN}\right|}^{2}<x\right), \end{array}$$

It is computed equation which illustrates the selected antenna index as

(A5) $$\begin{array}{cc} \mathrm{Pr}\left(i^{*}=i\right) & =\mathrm{Pr}\left(\overset{K}{\underset{j=1,j\neq i}{⋂}}{\left|h_{jF}\right|}^{2}<{\left|h_{iF}\right|}^{2}\right)\\ & =\int_{0}^{\infty }\prod_{j=1,j\neq i}^{K}\left[1-\mathrm{Pr}\left({\left|h_{jF}\right|}^{2}<x\right)\right]f_{{\left|h_{iF}\right|}^{2}}\left(x\right)\\ & =1/K. \end{array}$$

(A6) $$\begin{array}{c} F_{{\left|h_{iN}\right|}^{2}}\left(x\right)=1-e^{-\frac{x}{\Omega_{SN}}}\\ \;f_{{\left|h_{iN}\right|}^{2}}\left(x\right)=\frac{1}{\Omega_{SN}}e^{-\frac{x}{\Omega_{SN}}} \end{array}$$

(A7) $$\Omega_{1A}=1-e^{-\frac{\gamma_{2}}{\Omega_{SN}\left(a_{1}-a_{2}\gamma_{2}\right)}},$$

Using order statistics, the cumulative distribution function (CDF) and probability distribution function (PDF) of the channel gain can be written as

(A8) $$F_{{\left|h_{i^{*}F}\right|}^{2}}\left(y\right)=\sum_{k=0}^{K}\left(\begin{array}{c} K\\ k \end{array}\right){\left(-1\right)}^{k}e^{-\frac{kx}{\Omega_{SF}}},$$

(A9) $$f_{{\left|h_{i*F}\right|}^{2}}\left(x\right)=\sum_{k=1}^{K}\left(\begin{array}{c} K\\ k \end{array}\right)\frac{k{\left(-1\right)}^{k-1}}{\lambda_{SF}}e^{-\frac{kx}{\lambda_{SF}}},$$

(A10) $$\Omega_{1B}=\sum_{k=0}^{K}\left(\begin{array}{c} K\\ k \end{array}\right){\left(-1\right)}^{k}e^{-\frac{k\gamma_{2}}{\Omega_{SF}\left(b_{1}-b_{2}\gamma_{2}\right)}}.$$

(A11) $$\begin{array}{c} \Omega_{2}=\underset{\Omega_{2A}}{\underbrace{\mathrm{Pr}\left(\gamma_{i^{*}F}<\gamma_{2}\right)}}\underset{\Omega_{2B}}{\underbrace{\mathrm{Pr}\left(\gamma_{i^{*}N}^{x_{F}}\geq \gamma_{2},\gamma_{NF}<\gamma_{2}\right)}}, \end{array}$$

where $\Omega_{2A}=\Omega_{1B}$ can be re-expressed as

(A12) $$\Omega_{2B}=e^{-\frac{\gamma_{2}}{\Omega_{SN}\left(a_{1}-a_{2}\gamma_{2}\right)}}\left(1-e^{-\frac{\gamma_{2}}{\Omega_{NF}\rho }}\right).$$

Therefore substituting Equations (A7), (A10) and (A12) into (A1), the proof is completed. □

## Appendix B

**Proof** **of Proposition 2.** Starting on such analysis on ergodic capacity as below

(A13) $$C_{SiF}^{\left(II\right)}=\underset{I_{1}}{\underbrace{\frac{1}{2}\int_{0}^{\infty }\log_{2}\left(1+x\right)f_{X}\left(x\right)dx}}-\underset{I_{2}}{\underbrace{\frac{1}{2}\int_{0}^{\infty }\log_{2}\left(1+y\right)f_{Y}\left(y\right)dy}}$$

We first compute the ergodic capacity of each component as

(A14) $$\begin{array}{c} I_{1}=\frac{1}{2}\int_{0}^{\infty }\log_{2}\left(1+x\right)f_{X}\left(x\right)dx\\ \;\;\;\;\;\;\;=\frac{1}{2\ln2}\int_{0}^{\infty }\frac{1-F_{X}\left(x\right)}{1+x}\;dx \end{array}$$

(A15) $$\begin{array}{c} I_{2}=\frac{1}{2}\int_{0}^{\infty }\log_{2}\left(1+y\right)f_{Y}\left(y\right)dy\\ \;\;\;\;\;\;\;=\frac{1}{2\ln2}\int_{0}^{\infty }\frac{1-F_{Y}\left(y\right)}{1+y}\;dy \end{array}$$

Then we use PDF of X, Y and followed by some mathematical simplifications

(A16) $$\begin{array}{cc} F_{X}\left(x\right) & =\mathrm{Pr}\left({\left|h_{iF}\right|}^{2}\rho <x\right)\\ & =\mathrm{Pr}\left({\left|h_{iF}\right|}^{2}<\frac{x}{\rho }\right)\\ & =\int_{0}^{\frac{x}{\rho }}f_{{\left|h_{iF}\right|}^{2}}\left(x\right)dx\\ & =1-e^{-\frac{x}{\rho \Omega_{SF}}} \end{array}$$

(A17) $$\begin{array}{cc} F_{Y}\left(y\right) & =\mathrm{Pr}\left({\left|h_{iF}\right|}^{2}\rho \phi_{2}<y\right)\\ & =\mathrm{Pr}\left({\left|h_{iF}\right|}^{2}<\frac{y}{\phi_{2}\rho }\right)\\ & =\int_{0}^{\frac{y}{\rho \phi_{2}}}f_{{\left|h_{iF}\right|}^{2}}\left(y\right)dy\\ & =1-e^{-\frac{y}{\rho \phi_{2}\Omega_{SF}}} \end{array}$$

Then, it can be achieved that

(A18) $$\begin{array}{c} I_{1}=\frac{1}{2\ln2}\int_{0}^{\infty }\frac{1-(1-e^{-\frac{x}{\rho \Omega_{SF}}})}{1+x}dx\\ \;\;\;\;\;\;\;=\frac{1}{2\ln2}\int_{0}^{\infty }\frac{e^{-\frac{x}{\rho \Omega_{SF}}}}{1+x}dx \end{array}$$

(A19) $$\begin{array}{c} I_{2}=\frac{1}{2\ln2}\int_{0}^{\infty }\frac{1-(1-e^{-\frac{y}{\rho \phi_{2}\Omega_{SF}}})}{1+y}dy\\ \;\;\;\;\;\;\;=\frac{1}{2\ln2}\int_{0}^{\infty }\frac{e^{-\frac{y}{\rho \phi_{2}\Omega_{SF}}}}{1+y}dy \end{array}$$

Using ([39], Equation (3.352.4)), $I_{1}$ and $I_{2}$ are further given by

(A20) $$I_{1}=\frac{-e^{\frac{1}{\rho \Omega_{SF}}}}{2\ln2}\mathrm{Ei}\left(-\frac{1}{\rho \Omega_{SF}}\right)$$

(A21) $$I_{2}=\frac{-e^{\frac{1}{\rho \phi_{2}\Omega_{SF}}}}{2\ln2}\mathrm{Ei}\left(-\frac{1}{\rho \phi_{2}\Omega_{SF}}\right)$$

Finally, we obtain the derived expression for ergodic capacity in this mode

(A22) $$\begin{array}{cc} C_{iF}^{pro}= & \left\{C_{iF}\right\}\\ = & I_{1}-I_{2}\\ = & \frac{e^{\frac{1}{\rho \phi_{2}\Omega_{SF}}}}{2\ln2}\mathrm{Ei}\left(-\frac{1}{\rho \phi_{2}\Omega_{SF}}\right)-\frac{e^{\frac{1}{\rho \Omega_{SF}}}}{2\ln2}\mathrm{Ei}\left(-\frac{1}{\rho \Omega_{SF}}\right) \end{array}$$

where $\mathrm{Ei}\left(.\right)$ is the Exponential integrals function. □

## Appendix C

**Proof** **of Proposition 4.** Initially, it is defined that

(A23) $$\begin{array}{cc} C_{SiF}^{\left(II\right)} & =E\left\{C_{\mathrm{SiF}}\right\}\\ & =\frac{1}{2}\int_{0}^{\infty }\log_{2}\left(1+x\right)f_{X}\left(x\right)dx-\frac{1}{2}\int_{0}^{\infty }\log_{2}\left(1+y\right)f_{Y}\left(y\right)dy \end{array}$$

The CDF of *W* is given as [31] $F_{W}\left(w\right)=1-\exp\left(-\frac{w}{\Omega_{0}}\right){\sum_{i=0}^{K-1}\left(\frac{w}{\Omega_{0}}\right)}^{i}\frac{1}{i!}.$ For ease of computation, we denote $X=\frac{\rho }{2}\sum_{i=0}^{1}{\left|h_{iF}\right|}^{2}$, $Y=\frac{\phi_{2}\rho }{2}\sum_{i=0}^{1}{\left|h_{iF}\right|}^{2}.$ Then, the CDFs of these variables can be shown that

(A24) $$\begin{array}{cc} F_{X}\left(x\right) & =1-exp\left(-\frac{2x}{P_{S}\Omega_{SF}}\right){\sum_{i=0}^{1}\left(\frac{2x}{P_{S}\Omega_{SF}}\right)}^{i}\frac{1}{i!}\\ & =1-\left(1+\frac{2x}{P_{S}\Omega_{SF}}\right)exp\left(-\frac{2x}{P_{S}\Omega_{SF}}\right)\\ & =1-\left(1+ax\right)exp\left(-ax\right) \end{array}$$

(A25) $$\begin{array}{cc} F_{Y}\left(y\right) & =1-exp\left(-\frac{2x}{\phi_{2}\rho \Omega_{SF}}\right){\sum_{i=0}^{1}\left(\frac{2x}{\phi_{2}\rho \Omega_{SF}}\right)}^{i}\frac{1}{i!}\\ & =1-\left(1+\frac{2x}{\phi_{2}\rho \Omega_{SF}}\right)exp\left(-\frac{2x}{\phi_{2}\rho \Omega_{SF}}\right)\\ & =1-\left(1+bx\right)exp\left(-bx\right) \end{array}$$

where $a=\frac{2}{\rho \Omega_{SF}}$, $b=\frac{2}{\phi_{2}\rho \Omega_{SF}}$. After taking the derivative of $F_{X}\left(x\right)$ and $F_{Y}\left(y\right)$ the PDF of can be written as

(A26) $$f_{X}\left(x\right)=a^{2}xe^{-ax}$$

(A27) $$f_{Y}\left(y\right)=b^{2}ye^{-by}$$

Using the PDFs $f_{X}\left(x\right)$ and $f_{Y}\left(y\right)$. $C_{iF}^{pro}$ can be rewritten as follows

(A28) $$C_{SiF}^{\left(II\right)}=\frac{1}{2}\left\{a^{2}\int_{0}^{\infty }\log_{2}\left(1+x\right)x\;e^{-ax}dx\right\}-\frac{1}{2}\left\{b^{2}\int_{0}^{\infty }\log_{2}\left(1+y\right)y\;e^{-by}dy\right\}$$

Using $\log_{a}\left(x/y\right)=\;\log_{a}x-\;\log_{a}y$. $C_{SiF}^{\left(II\right)}$ can be written as

(A29) $$C_{SiF}^{\left(II\right)}=\underset{I_{1}}{\underbrace{\frac{1}{2\ln(2)}\left\{a^{2}\int_{0}^{\infty }\ln\left(1+x\right)x\;e^{-ax}dx\right\}}}-\underset{I_{2}}{\underbrace{\frac{1}{2\ln(2)}\left\{b^{2}\int_{0}^{\infty }\ln\left(1+y\right)y\;e^{-by}dy\right\}}}$$

(A30) $$I_{1}=\frac{1}{2\ln(2)}\left\{a^{2}\int_{0}^{\infty }\ln\left(1+x\right)x\;e^{-ax}dx\right\}$$

Putting $u=ax\Rightarrow \frac{1}{a}u=x\Rightarrow \frac{1}{a}du=dx$. $I_{1}$, can be written as

(A31) $$\begin{array}{c} I_{1}=\frac{1}{2\ln(2)}\left\{a^{2}\int_{0}^{\infty }\frac{1}{a^{2}}\ln\left(1+u\right)\;ue^{-u}du\right\}\\ \;\;\;\;\;\;\;=\frac{1}{2\ln(2)}\left\{\int_{0}^{\infty }\ln\left(1+u\right)\;ue^{-u}du\right\} \end{array}$$

Using ([39], Equation (4.337.5*)), $I_{1}$ given by

(A32) $$\begin{array}{c} I_{1}=\frac{1}{2\ln(2)}\left\{ae^{a}\mathrm{Ei}\left(-a\right)-e^{a}\mathrm{Ei}\left(-a\right)+1\right\}\\ \;\;\;\;\;\;\;=\frac{1}{2\ln(2)}\left\{ae^{a}\mathrm{Ei}\left(-a\right)\left(a-1\right)+1\right\}. \end{array}$$

Similarly, $I_{2}=\frac{1}{2\ln(2)}\left\{be^{b}\mathrm{Ei}\left(-b\right)\left(b-1\right)+1\right\}$, $C_{SiF}^{\left(II\right)}$ can be written as

(A33) $$\begin{array}{cc} C_{SiF}^{\left(II\right)} & =\frac{1}{2\ln(2)}\left\{ae^{a}\mathrm{Ei}\left(-a\right)-e^{a}\mathrm{Ei}\left(-a\right)+1-\left(be^{b}\mathrm{Ei}\left(-b\right)\left(b-1\right)+1\right)\right\}\\ & =\frac{1}{2\ln(2)}\left\{ae^{a}\mathrm{Ei}\left(-a\right)-e^{a}\mathrm{Ei}\left(-a\right)-be^{b}\mathrm{Ei}\left(-b\right)\left(b-1\right)\right\}. \end{array}$$

It completes the proof. □
